# Inflamação e Prognóstico em Insuficiência Cardíaca Aguda: Existe um Papel para o Índice de Imunoinflamação Sistêmica?

**DOI:** 10.36660/abc.20240286

**Published:** 2024-07-11

**Authors:** Humberto Villacorta

**Affiliations:** 1 Universidade Federal Fluminense Niterói RJ Brasil Universidade Federal Fluminense, Niterói, RJ – Brasil

**Keywords:** Insuficiência Cardíaca, Inflamação, Biomarcadores, Prognóstico, Hospitalização, Endotoxinas

A insuficiência cardíaca (IC) é um importante problema de saúde, com altas taxas de morbidade e mortalidade.^1^ Pacientes com IC são frequentemente hospitalizados. A inflamação desempenha um papel na fisiopatologia da IC e várias citocinas, como o fator de necrose tumoral (TNF), o fator de crescimento transformador-ß (TGF-ß) e as interleucinas 6 e 1, estão elevadas na IC.^2^ Na IC aguda descompensada (ICAD) os desencadeadores desta cascata inflamatória são secundários à ativação neuro-hormonal e ao estresse oxidativo, mas, além disso, há evidências de elevada translocação bacteriana ou de endotoxinas, devido a edema intestinal ou hipoperfusão relativa.^2,3^ A medição de citocinas não é apropriada para a prática clínica diária e a proteína C reativa (PCR),^4,5^ outro biomarcador de inflamação, é geralmente escolhida para avaliar a inflamação. No entanto, é necessário um marcador mais robusto e conveniente.

**Figura 1:**
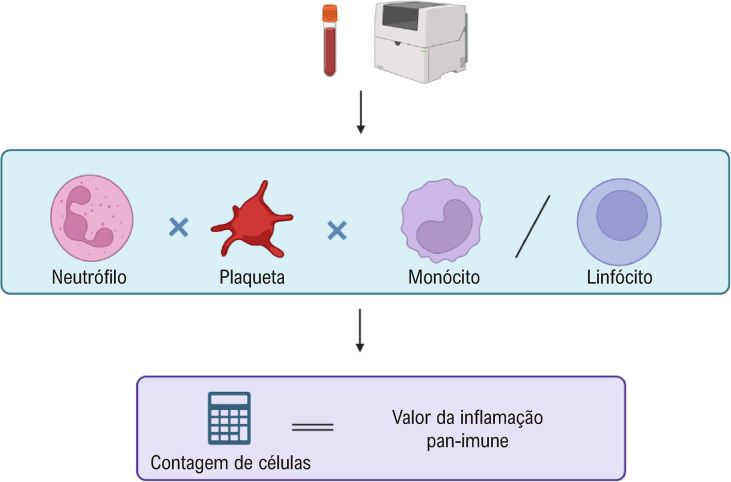
– O valor da inflamação pan-imune é um novo biomarcador, associado à inflamação sistêmica, calculado pela multiplicação da contagem de neutrófilos, plaquetas e monócitos, dividida pela contagem de linfócitos.

Um novo marcador de inflamação foi introduzido recentemente. O valor pan-imune-inflamatório (PIV) é calculado a partir dos componentes imunoinflamatórios do sangue periférico, incluindo contagens de neutrófilos, plaquetas, monócitos e linfócitos, conforme mostrado na Figura 1. O PIV demonstrou ser prognóstico em alguns distúrbios cardiovasculares e em doenças não cardiovasculares, como câncer e doença renal avançada.^6-10^

Nesta edição dos Arquivos Brasileiros de Cardiologia, um estudo original apresenta uma investigação sobre o valor prognóstico do PIV em pacientes com ICAD.^11^ Os resultados do estudo sugerem que níveis mais elevados de PIV na admissão estão associados ao aumento da mortalidade por todas as causas a curto e longo prazo em pacientes com ICAD. Um ponto forte notável do estudo é o foco em um novo biomarcador, o PIV, que oferece uma avaliação abrangente da inflamação ao incorporar múltiplos componentes imunoinflamatórios. Esta abordagem fornece uma compreensão mais sutil do estado inflamatório em pacientes com IC em comparação com marcadores de componente único. Ao considerar vários aspectos da resposta imune, o PIV pode oferecer maior precisão prognóstica e valor preditivo. É digno de nota que o valor prognóstico do PIV neste estudo foi independente do peptídeo natriurético pró-tipo B N-terminal (NT-proBNP) e da PCR.

Algumas limitações do estudo^11^ já foram abordadas pelos autores e devem ser consideradas na interpretação dos resultados do estudo. Em primeiro lugar, o desenho retrospectivo do estudo e a configuração de centro único podem introduzir viés de seleção e limitar a generalização dos resultados. Além disso, o tamanho relativamente pequeno da amostra de 409 pacientes levanta preocupações sobre o poder estatístico e a robustez dos resultados. Estudos multicêntricos maiores são necessários para validar a utilidade prognóstica do PIV em diversas populações de pacientes.

Além disso, embora o estudo^11^ demonstre uma associação entre o PIV e os resultados de mortalidade, os mecanismos subjacentes que impulsionam esta relação permanecem obscuros. O estudo não fornece informações sobre as vias específicas pelas quais a desregulação imunoinflamatória contribui para resultados adversos em pacientes com IC. Pesquisas futuras devem ter como objetivo elucidar os mecanismos fisiopatológicos que ligam o PIV ao prognóstico da IC, potencialmente através de estudos mecanísticos ou validação de biomarcadores em modelos animais.

Apesar dessas limitações, este estudo^11^ traz informações novas e relevantes. Até onde sabemos, apenas um estudo anterior avaliou o PIV na ICAD e, portanto, o presente estudo acrescenta informações neste cenário, onde os dados são escassos.^12^ Esperamos estudos futuros com este novo biomarcador em populações maiores.
