# Supplementary material for: Inflamação e Prognóstico em Insuficiência Cardíaca Aguda: Existe um Papel para o Índice de Imunoinflamação Sistêmica?
Source: Arq Bras Cardiol. 2024 Jul 11;121(6):e20240286. [Article in Portuguese] doi: 10.36660/abc.20240286 (PMC12080625; doi:10.36660/abc.20240286)
Supplement: Translation

# Translation: Inflammation and Prognosis in Acute Heart Failure: Is There a Role for Pan-Immune-Inflammation Value?

**DOI:** 10.36660/abc.20240286i

**Authors:** Humberto Villacorta

**Affiliations:** 1 Brazil Universidade Federal Fluminense, Niterói, RJ – Brazil

**Keywords:** Heart Failure, Inflammation, Biomarkers, Prognosis, Hospitalization, Endotoxins,Hospitalization, Endotoxins

Heart failure (HF) is a major health problem, with high morbidity and mortality rates.^1^ Patients with HF are frequently hospitalized. Inflammation plays a role in the pathophysiology of HF and several cytokines, such as tumor necrosis factor (TNF), transforming growth factor-ß (TGF-ß), and interleukins 6 and 1, are elevated in HF.^2^ In acute decompensated HF (ADHF) the triggers of this inflammatory cascade are secondary to neurohormonal activation and oxidative stress, but in addition, there is evidence for elevated bacterial or endotoxin translocation, due to gut edema or relative hypoperfusion.^2,3^ The measurement of cytokines is not appropriate for day-to-day clinical practice and C-reactive protein (CRP),^4,5^ another biomarker of inflammation, is usually chosen to assess inflammation. However, a more robust and convenient marker is warranted.

A new marker of inflammation has been recently introduced. Pan-immune-inflammation value (PIV) is calculated from peripheral blood immune-inflammatory components, including neutrophil, platelet, monocyte, and lymphocyte counts, as shown in Figure 1. PIV has been shown to be prognostic in some cardiovascular disorders and also in non-cardiovascular diseases, such as cancer and advanced renal disease.^6-10^

**Figure 1:** – The pan-immune-inflammation value is a new biomarker, associated with systemic inflammation, calculated by multiplying neutrophil, platelet, and monocyte counts, divided by the lymphocyte count.

In this issue of *Arquivos Brasileiros de Cardiologia*, an original study presents an investigation into the prognostic value of PIV in patients with ADHF.^11^ The study’s findings suggest that higher PIV levels at admission are associated with increased short and long-term all-cause mortality in patients with ADHF. One notable strength of the study is its focus on a novel biomarker, PIV, which offers a comprehensive assessment of inflammation by incorporating multiple immune-inflammatory components. This approach provides a more nuanced understanding of the inflammatory status in HF patients compared to single-component markers. By considering various aspects of the immune response, PIV may offer improved prognostic accuracy and predictive value. Of note, the prognostic value of PIV in this study was independent of N-terminal pro-B-type natriuretic peptide (NT-proBNP) and CRP.

Some limitations of the study^11^ have already been addressed by the authors and should be considered when interpreting the study findings. Firstly, the study’s retrospective design and single-center setting may introduce selection bias and limit the generalizability of the results. Additionally, the relatively small sample size of 409 patients raises concerns about statistical power and the robustness of the findings. Larger, multicenter studies are needed to validate the prognostic utility of PIV in diverse patient populations.

Furthermore, while the study^11^ demonstrates an association between PIV and mortality outcomes, the underlying mechanisms driving this relationship remain unclear. The study does not provide insights into the specific pathways through which immune-inflammatory dysregulation contributes to adverse outcomes in HF patients. Future research should aim to elucidate the pathophysiological mechanisms linking PIV to HF prognosis, potentially through mechanistic studies or biomarker validation in animal models.

Despite these limitations, this study^11^ brings new and relevant information. To the best of our knowledge, only one previous study evaluated PIV in ADHF, and, therefore, the present study adds information in this scenario, where data is scarce.^12^ We look forward to future studies with this new biomarker in larger populations.
